# Changes in Gait Speed Vary Widely after the Use of Antiparkinson Medication in Persons with Parkinson's Disease

**DOI:** 10.1002/mdc3.70169

**Published:** 2025-06-12

**Authors:** Anson B. Rosenfeldt, Cielita Lopez‐Lennon, Erin Suttman, Amy Elizabeth Jansen, Eric Zimmerman, Courtney Miller, Rachel Lovati, Hubert H. Fernandez, Peter B. Imrey, Leland E. Dibble, Jay L. Alberts

**Affiliations:** ^1^ Department of Biomedical Engineering, Lerner Research Institute Cleveland Clinic Cleveland Ohio USA; ^2^ Department of Physical Therapy and Athletic Training University of Utah Salt Lake City Utah USA; ^3^ Center for Neurological Restoration, Neurological Institute Cleveland Clinic Cleveland Ohio USA; ^4^ Department of Rehabilitation Services, Neurological Institute Cleveland Clinic Cleveland Ohio USA; ^5^ Department of Quantitative Health Sciences, Lerner Research Institute Cleveland Clinic Cleveland Ohio USA; ^6^ Mellen Center for Multiple Sclerosis Treatment and Research, Neurological Institute Cleveland Clinic Cleveland Ohio USA; ^7^ Cleveland Clinic Lerner College of Medicine of Case Western Reserve University Cleveland Ohio USA

**Keywords:** antiparkinson medication, dopamine, gait speed, Parkinson's disease, Six‐Minute Walk Test

## Abstract

**Background:**

Antiparkinson medications are effective in improving Parkinson's disease (PD) motor symptoms such as bradykinesia, tremor, and rigidity. The impact of antiparkinson medication on gait speed is less clear.

**Objectives:**

The primary aim was to determine the effects of antiparkinson medication on gait speed in PD. The secondary aim was to identify characteristics associated with the magnitude of response.

**Methods:**

Two hundred fifty individuals with PD completed the Six‐Minute Walk Test *on* and *off* medication, on separate days, in a randomized order. A paired *t*‐test and linear regression were used to assess *on*‐ to *off*‐medication gait speed differences and their relationships with potential correlates. Using previously established values, changes in gait speed were characterized as small (0.06–0.13 m/s), moderate (0.14–0.21 m/s), and large (>0.22 m/s). Corresponding summary characteristics were provided for each classification.

**Results:**

Mean (standard deviation) gait speed significantly increased from 1.33 (0.31) to 1.40 (0.28) m/s from *off* to *on* medication. Longer disease duration, greater clinical ratings of gait and postural instability, slower functional mobility, and higher levodopa equivalent daily dose were associated with larger increases in gait speed. In sum, 131 (52%) participants experienced no improvement or an improvement that did not meet the threshold for small responder. Of the 119 (48%) participants identified as responders, 60 were classified as small, 28 moderate, and 31 large responders.

**Conclusions:**

Overall, gait speed improved with antiparkinson medication; however, individual responses varied. Gait dysfunction in PD may be caused by impairment of multiple neural circuits, some of which are less responsive to antiparkinson medication.

Gait dysfunction is a cardinal symptom of Parkinson's disease (PD). Early in the disease process, gait dysfunction typically manifests in decreased arm swing, shortened step length, and slower gait speed.^1^ As PD progresses, more severe gait impairments emerge, including festinating gait patterns with freezing episodes and falls.^1^ Antiparkinson medications are a first‐line treatment of PD and are effective in improving PD motor symptoms such as bradykinesia, tremor, and rigidity.^2^, ^3^ However, the impact of medication on gait and postural impairment is less clear. Aspects of gait related to speed and amplitude such as gait speed, stride length, and arm swing appear most responsive to antiparkinson medication.^4^, ^5^, ^6^, ^7^, ^8^ Other temporal and postural aspects of gait such as cadence, double limb support time, and gait variability appear less responsive to medication.^4^, ^5^, ^6^, ^7^, ^8^ Limitations of the previous work examining the impact of antiparkinson medication on gait speed include small sample sizes^4^, ^5^, ^7^, ^8^ and nonrandomized testing order with the *off*‐medication testing session frequently preceding the *on*‐medication testing,^4^, ^5^, ^6^, ^8^ rendering the results susceptible to biases such as familiarization effects.

Although antiparkinson medication generally has a positive impact on PD symptoms, individual responses to antiparkinson medication can vary widely.^9^, ^10^ Current best practice in PD medication therapy involves adjusting antiparkinson medication to mitigate individual symptoms while concomitantly limiting associated side effects.^11^ Significant gaps remain regarding which individuals with PD are most likely to experience improvements in gait with antiparkinson medication therapy. Providing clinicians with insight into *who* may benefit from antiparkinson medication, and *to what extent* they benefit, will aid in clinical decision‐making and facilitate setting appropriate expectations for individuals with PD.

The primary aim of this project was to evaluate the effect of antiparkinson medication on gait speed in a large cohort of individuals with PD. As part of a randomized clinical trial, individuals with PD completed the Six‐Minute Walk Test (6MWT) in the *on*‐ and *off*‐medication states, in random order, on 2 separate days. It was hypothesized that antiparkinson medication would have a positive effect on gait speed. The secondary aim was to determine demographic or performance variables associated with the magnitude of gait speed response to antiparkinson medication. An enhanced understanding of factors related to greater improvement in gait speed has the potential to refine the role of antiparkinson medication.

## Patients and Methods

Data were collected as part of a large, randomized clinical trial, CYClical Lower Extremity Exercise Trial for Parkinson's disease (CYCLE‐II), conducted at the Cleveland Clinic (Cleveland, OH) and the University of Utah (Salt Lake City, UT). Details of the study protocol have been previously published^12^ and are summarized subsequently. Prior to participation, participants completed the informed consent process approved by the institutional review boards (IRB) of the Cleveland Clinic (IRB of record) and the University of Utah.

### Participants

Adults with idiopathic PD with Hoehn and Yahr stages I to III on a stable dose of antiparkinson medication were included. Exclusion criteria were participation in pharmaceutical or behavioral disease–modifying PD‐related trial, diagnosis of dementia or other neurocognitive impairment compromising the ability to provide informed consent, history of deep brain stimulation (DBS) or focused ultrasound, neurological disease other than PD, and cardiac arrhythmia. The study involved additional safety and feasibility screening to ensure individuals were safe to participate in moderate‐ to high‐intensity aerobic exercise.

### Assessment Protocol

Participants completed 2 assessments: (1) *on* medications, 1 h after intake of their antiparkinson medication, and (2) *off* medications, practically defined as withholding antiparkinson medication for 12 h. The order of the assessments was randomized, and testing sessions were completed at least 24 h apart. Demographic data collection and medication reconciliation were performed at the first assessment.

### Outcomes

The 6MWT is a reliable measure of gait speed and functional endurance in adults with neurological disease.^13^, ^14^ The 6MWT was administered on a 20‐m path, 1.8‐m‐wide. Participants were instructed to walk as far as possible during the 6 min without running or jogging.^15^ Distance walked was recorded; gait speed (m/s) was calculated from traversed distance divided by time.

Established important differences in gait speed for PD were used to classify the degree of gait improvement.^16^ An increase in gait speed of 0.06 to 0.13 m/s was considered a small important difference, 0.14 to 0.21 m/s moderate, and ≥0.22 m/s large. Those who experienced a change in gait speed <0.06 were classified as clinical non‐responders.

Additional outcome metrics included the Movement Disorder Society–Unified Parkinson's Disease Rating Scale Motor III (MDS‐UPDRS III) and the Timed Up and Go (TUG) test. The MDS‐UPDRS III was used to assess PD global motor symptoms.^17^ Subscales of tremor (items 3.15–3.18), bradykinesia (items 3.4–3.8, 3.14), rigidity (item 3.3), and postural instability and gait dysfunction (PIGD, items 3.9–3.13) were calculated from the MDS‐UPDRS III. The TUG is a measure of functional mobility where the individual stands from a seated position on a chair, ambulates 3 m, turns, returns to the chair, and sits.^18^

### Statistical Analysis

Demographic and outcome variables are summarized as mean (SD [standard deviation]) or N (%). In descriptive summaries, participants were categorized by the change in 6MWT speed from the *off*‐ to *on*‐medication state based on the established important differences defined previously. The levodopa equivalent daily dose (LEDD) was calculated based on established conversion values.^19^, ^20^, ^21^

For the primary aim, a paired *t*‐test was used to assess the difference in gait speed during the 6MWT *on* versus *off* medication. Secondarily, linear regression models were developed to determine what characteristics were related to the magnitude of medication response. An initial model including basic demographics (age, sex, disease duration) and LEDD was developed to predict change in gait speed from the *off*‐ to *on*‐medication state. Then, for the TUG duration, MDS‐UPDRS III total score, and MDS‐UPDRS III subscores of PIGD, tremor, bradykinesia, and rigidity (all *off* medication), separate regression models were developed by additionally including the variable of interest along with age, sex, years of PD, and LEDD to control for potential confounding.

Continuously measured predictors were incorporated in regression models as continuous linear covariates. The assumption of normality was assessed visually using Q–Q plots. All statistical tests of significance were conducted using a 0.05 level of significance. All statistical analyses were conducted using RStudio, version 2024.04.2, and R, version 4.4.0.

## Results

A total of 256 individuals with PD were enrolled in the CYCLE‐II trial. Five individuals withdrew during the baseline assessments and 1 individual had an invalid 6MWT. The remaining 250 individuals were included in the primary analysis. Of these, 1 participant had an unknown LEDD and was excluded from the regression analysis. Demographics and baseline clinical and gait metrics (TUG and 6MWT) as a function of medication are provided in Table 1.

**TABLE 1:** Demographics and baseline clinical and gait metrics by category of six‐minute walk test speed response among 250 individuals with Parkinson's disease

	Overall (n = 250)	Non‐responder (n = 131)	Small responder (n = 60)	Moderate responder (n = 28)	Large responder (n = 31)
Age (yr)	64.1 (8.3)	64.5 (8.3)	65.1 (7.7)	58.8 (8.4)	64.9 (8.3)
Sex, male	166 (66.4%)	88 (67.2%)	38 (63.3%)	19 (67.9%)	21 (67.7%)
Disease duration (yr)	4.7 (4.0)	4.0 (3.5)	5.0 (4.0)	4.1 (3.0)	7.1 (5.3)
LEDD^a^	673 (395)	584 (350)	720 (353)	676 (336)	952 (542)
MDS‐UPDRS III, total score					
*Off* medication	37.7 (14.8)	37.6 (14.7)	35.5 (14.9)	39.9 (11.8)	40.3 (16.9)
*On* medication	29.3 (13.3)	31.1 (13.2)	27.2 (12.6)	27.0 (13.0)	27.6 (15.0)
PIGD subscale of the MDS‐UPDRS III					
*Off* medication	4.4 (3.3)	4.2 (3.1)	3.8 (2.9)	4.1 (2.6)	6.5 (4.6)
*On* medication	3.3 (2.9)	3.5 (2.9)	3.0 (2.8)	2.7 (2.4)	4.0 (3.5)
Tremor subscale of the MDS‐UPDRS III					
*Off* medication	7.5 (5.6)	7.5 (5.4)	7.3 (5.5)	8.6 (4.9)	6.9 (7.0)
*On* medication	5.0 (4.3)	5.4 (4.3)	5.2 (4.1)	4.8 (4.1)	3.1 (4.2)
Bradykinesia subscale of the MDS‐UPDRS III					
*Off* medication	16.7 (7.0)	16.6 (6.8)	15.8 (7.4)	18.5 (5.9)	17.2 (7.8)
*On* medication	13.5 (6.6)	14.4 (6.4)	12.0 (6.3)	13.1 (6.6)	13.0 (7.5)
Rigidity subscale of the MDS‐UPDRS III					
*Off* medication	6.8 (4.0)	7.0 (3.8)	6.3 (4.3)	6.5 (3.8)	7.4 (4.0)
*On* medication	5.5 (3.7)	5.9 (3.7)	5.2 (3.7)	4.6 (3.1)	5.6 (3.7)
Timed Up and Go Test (s)					
*Off* medication	10.7 (3.7)	10.4 (3.5)	10.0 (2.2)	10.4 (2.1)	13.3 (6.2)
*On* medication	10.2 (3.4)	10.4 (3.9)	9.7 (1.9)	9.1 (1.6)	10.8 (4.1)
Six‐Minute Walk Test (m/s)					
*Off* medication	1.33 (0.31)	1.39 (0.31)	1.33 (0.23)	1.34 (0.24)	1.05 (0.35)
*On* medication	1.40 (0.28)	1.36 (0.31)	1.42 (0.22)	1.50 (0.24)	1.44 (0.27)

*Note*: Data are presented as mean (SD) or N (%).

Abbreviations: LEDD, levodopa equivalent daily dose; MDS‐UPDRS III, Movement Disorder Society–Unified Parkinson's Disease Rating Scale Motor III; PIGD, postural instability and gait dysfunction.

^a^ N = 249, LEDD dose missing in 1 participant.

### Antiparkinson Medication Improves Gait Speed

**FIG. 1:**
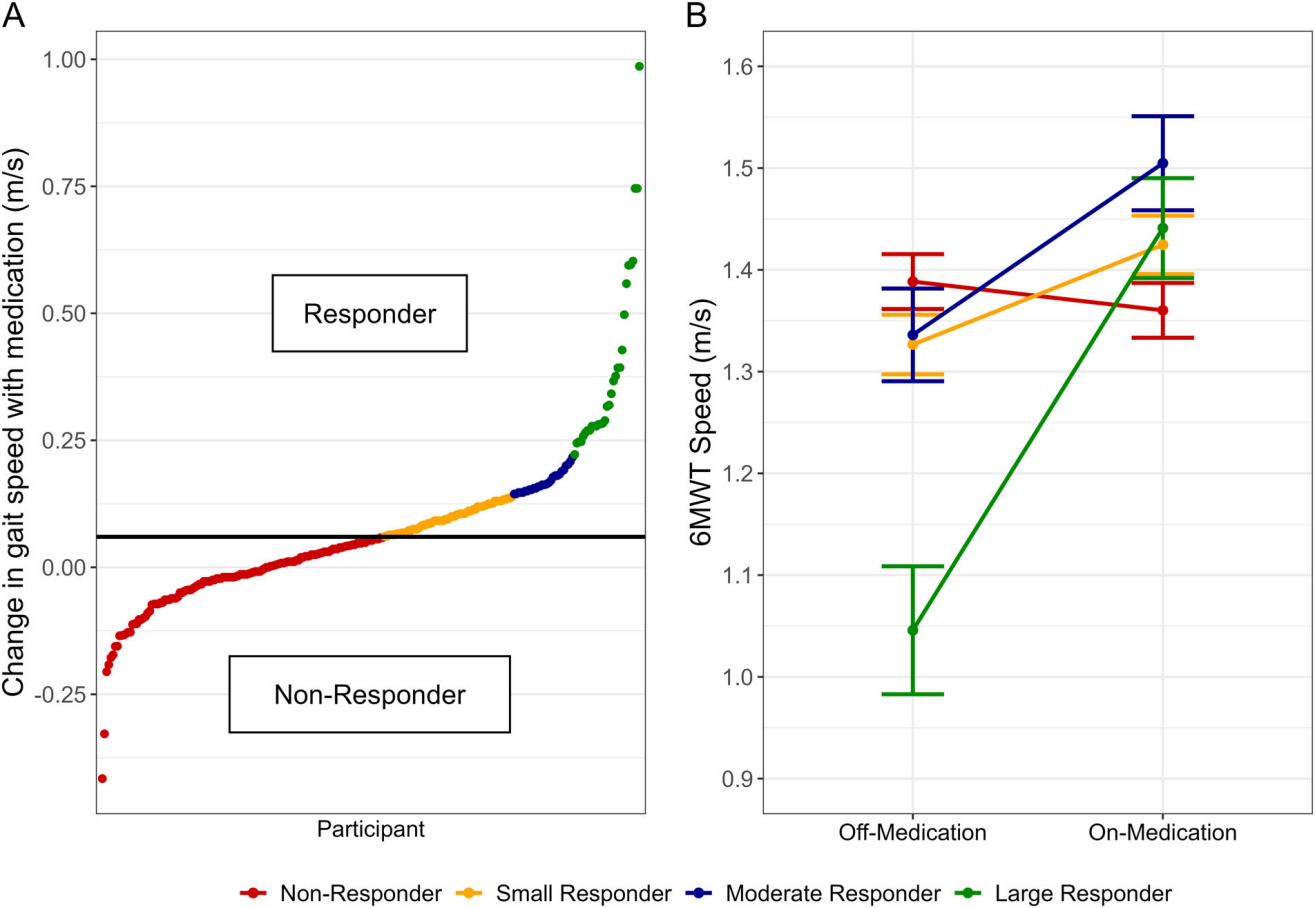
(**A**) Individual change in the 6MWT (Six‐Minute Walk Test) from the *off*‐ to the *on*‐medication state (N = 250). The horizonal line at 0.06 m/s denotes the small important difference, and a negative value indicates a slowing of gait in the *on*‐medication state. Those who experienced a change in gait speed of <0.06 m/s were considered non‐responders (N = 131 [52%], red). Of the 119 (48%) participants identified as responders, 60 were classified as small (increase of 0.06–0.13 m/s, yellow), 28 as moderate (0.14–0.21 m/s, blue), and 31 as large (≥0.22 m/s, green) responders. (**B**) Mean (±SE [standard error]) change in gait speed (m/s) from the *off*‐ to the *on*‐medication state during the Six‐Minute Walk Test (6MWT) in 250 individuals with PD (Parkinson's disease) classified by responder groups.

Overall, there was a significant improvement of 0.07 m/s in gait speed with medication. Gait speed during the 6MWT increased from 1.33 (0.31) to 1.40 (0.28) m/s from *off* to *on* antiparkinson medication (*P* < 0.001, standardized difference [Hedges' g] = 0.26). However, this overall improvement conceals wide individual response variability. One hundred thirty‐one (52%) participants experienced worsening in gait speed or an improvement in the on‐medication state that did not meet the small important difference threshold. The remaining 119 (48%) participants met or exceeded the criterion for small improvement in gait speed. Figure 1A shows the individual changes in gait speed in response to medication.

### Correlates of Individual Responses

In the initial regression model containing only demographic (age, sex, disease duration) and medication control variables, higher LEDD (coefficient [95% confidence interval, CI] = 0.01 [0.004, 0.015] m/s per 100 mg) and longer disease duration (coefficient [95% CI] = 0.01 [0.004, 0.014] m/s per year PD) were each associated with greater improvement in gait speed (*P* < 0.001 for both). Age and sex were not significantly related to change in gait speed (*P* = 0.66 and 0.14, respectively).

When demographics and LEDD were controlled, a larger MDS‐UPDRS III PIGD subscore was associated with greater improvement in gait speed (coefficient [95% CI] = 0.01 [0.003, 0.015] m/s per point, *P* = 0.004). Additionally, longer TUG duration was associated with larger improvements in gait speed (coefficient [95% CI] = 0.01 [0.005, 0.015] m/s change per second, *P* < 0.001). The MDS‐UPDRS III total score and subscores of tremor, bradykinesia, and rigidity were not significantly associated with change in gait speed.

### Differences among 6MWT Medication Response‐Level Categories

Overall, 60 (24%) individuals were classified as small responders, 28 (11%) as moderate responders, and 31 (12%) as large responders, along with the 131 (52%) non‐responders noted earlier. Figure 1B provides the magnitudes of change in gait speed for the 4 subgroups from the *off*‐ to *on*‐medication state.

The associations of *off*‐ to *on*‐medication differences with other variables are presented in Table 1 by comparing summary descriptive statistics across the response‐level categories. Based on the MDS‐UPDRS III total score, the non‐, small, moderate, and large responders improved by 17%, 23%, 32%, and 32%, respectively, after antiparkinson medication (Table 1). All groups experienced the largest percentage improvements in the tremor domain (28%–55%). Changes in the TUG mirrored 6MWT performance, with the non‐responder group experiencing no change in TUG time with medication and the large responder group experiencing a 19% reduction in TUG time.

## Discussion

As hypothesized and consistent with previous reports,^22^ antiparkinson medication resulted in an increase in gait speed in the overall sample of individuals with PD. The sample average increase in gait speed was 0.07 m/s, which exceeds the established threshold for the small important difference in change in gait speed in individuals with PD.^16^ The large sample size and the randomized testing order in the current study provide further evidence that antiparkinson medication, on average, improves gait speed in individuals with PD. However, improvement in gait speed was not ubiquitous. In fact, in the on‐medication state, over half of participants experienced a slowing of gait or an improvement that did not meet the small important difference threshold. Those who experienced the greatest improvements in gait speed in the *on*‐medication state had longer disease durations, more pronounced clinical ratings of gait and postural instability, slower functional mobility, and higher LEDD.

Diagnostic criterion and pharmaceutical trials in PD frequently consider an improvement of about 20% to 30% a meaningful response to medication.^23^, ^24^ Overall, the cohort in this study experienced a 22% improvement in MDS‐UPDRS III scores, suggesting the sample overall was medically optimized. The overall improvement was driven by the tremor subscale, which improved by 33%. Arguably, the visibility and ease of tremor assessment along with its responsiveness to antiparkinson medication^25^ may contribute to its medical optimization compared to other cardinal motor symptoms. The change in MDS‐UPDRS III varied among groups. The large responder group experienced a 32% reduction in mean total MDS‐UPDRS III score, with every subscale achieving a 20% or greater improvement. However, the non‐responder group experienced a 17% reduction in mean MDS‐UPDRS III score, with only the tremor subscale exceeding a 20% reduction. It is possible that those with less robust responses in clinical scales were not medically optimized and have the potential to experience greater improvement in both clinical and gait metrics with medication refinement.

In the *on*‐medication state, only the moderate responder group approached the gait speed of 1.5 to 1.6 m/s achieved by healthy adults aged 60 to 69 years during the 6MWT.^26^ The slower gait speed of PD patients *on* antiparkinson medication compared to their age‐matched peers suggests that PD is not purely a disease of dopamine deficiency but rather a multifactorial disease that adversely impacts other neural circuitry involved in controlling gait and posture.^27^, ^28^ Historically hallmarked by dopaminergic depletion, it is now accepted that PD adversely impacts noradrenergic, glutamatergic, serotonergic, and cholinergic cells, the latter of which appear to play a key role in PD‐related gait dysfunction.^29^, ^30^ Specifically, cholinergic degeneration of the pedunculopontine nucleus reduces connectivity between the cerebellum, thalamus, and regions of the frontal cortex, resulting in gait and balance dysfunction in human and primate models of PD.^28^, ^31^, ^32^ The involvement of multiple neural circuits controlling gait in PD is further supported by a recent study reporting contrasting changes in gait variability with DBS compared to antiparkinson medication,^33^ again suggesting both dopaminergic and nondopaminergic gait control in PD. Collectively, data indicate that the treatment of gait dysfunction in PD should reach beyond dopamine therapies to better target gait impairments.

Individuals in this cross‐sectional study had predominantly mild–moderate PD. Because dopaminergic medication generally becomes less effective with severe disease,^9^ further studies should be conducted on the role of dopaminergic medication in those with greater disease burden. Although clinician‐rated freezing of gait (FOG) is included in the MDS‐UPDRS III PIGD subscale, FOG episodes were not systematically monitored during the 6MWT. Due to the mixed reports of the impact of antiparkinson medication on FOG,^34^ future studies evaluating the impact of antiparkinson medication on gait should note the frequency and duration of FOG events. It can be argued that the 6MWT is a test of gait endurance rather than a shorter test of gait speed such as the Ten‐Meter Walk Test. In a study of over 300 people with PD, gait speed from the comfortable‐paced Ten‐Meter Walk Test and the 6MWT was strongly correlated (r = 0.75),^35^ and the authors concluded that providers may select either one to obtain information relating to comfortable gait speed. Finally, the observed improvement among large responders cannot be attributed only to benefit from their medication but, because the groups were defined by differences between repeated 6MWT results, also includes an additional contribution from 6MWT measurement variability.

Overall, gait speed in PD was positively impacted by antiparkinson medications. Those who experienced the greatest improvement in gait speed in the *on*‐medication state had longer disease durations, more pronounced clinical ratings of gait and postural instability, slower functional mobility, and higher LEDD. Over half of participants experienced a worsening or limited improvement in gait speed that did not meet the small important difference in the *on*‐medication state, suggesting that dopamine deficiency is not the only cause of gait impairment in PD. Continued work on the neurophysiology of gait impairment in PD is needed to unravel the effects of pharmacological and surgical interventions on PD gait.

## Author Roles

(1) Research project: A. Conception, B. Organization, C. Execution, D. Participant recruitment; (2) Statistical analysis: A. Design, B. Execution, C. Review and critique; (3) Manuscript preparation: A. Writing of the first draft, B. Review and critique.

A.B.R.: 1B, 1C, 2C, 3A

C.L.‐L.: 1C, 1D, 3B

E.S.: 1C, 1D

A.E.J.: 1C, 1D, 3B

E.Z.: 2B, 2C

C.M.: 1D, 3B

R.L.: 1D, 3B

H.H.F.: 1D, 3B

P.B.I.: 2A, 2C, 3B

L.E.D.: 1B, 1C, 3B

J.L.A.: 1A, 1B, 1C, 2C, 3B

## Disclosures

**Ethical Compliance Statement**: This study was approved by the institutional review boards of the Cleveland Clinic (IRB of record) and the University of Utah. Prior to initiation of the study protocol, all participants completed the informed consent process. We confirm that we have read the journal's position on issues involved in ethical publication and affirm that this work is consistent with those guidelines.

**Funding Sources and Relevant Conflicts of Interest**: This study is supported by the National Institute of Neurological Disorders and Stroke of the National Institutes of Health (2R01NS073717). The content is solely the responsibility of the authors and does not necessarily represent the official views of the National Institutes of Health. The authors declare no conflicts of interest relevant to this work.

**Financial Disclosures for the Previous 12 Months:** J.L.A. is a consultant for Qr8 Health and Ceraxis. J.L.A. and A.B.R. have authored intellectual property associated with augmented and virtual reality technology that has been licensed to Stroll Ltd and Elm Park Labs. A.B.R. and J.L.A. received funds for other projects from sources, including the Department of Defense, National Institutes of Health, and the Michael J. Fox Foundation. P.B.I. received funds for other projects from sources including the National Institutes of Health and Arthritis Foundation. L.E.D. received funds for other projects from sources, including the PAC‐12 Athletic Conference, Department of Defense, National Institutes of Health, and University of Utah for Intramural grants.

## References

[1] Mirelman A , Bonato P , Camicioli R , et al. Gait impairments in Parkinson's disease. Lancet Neurol 2019;18(7):697–708. 10.1016/S1474-4422(19)30044-4. 30975519

[2] Frequin HL , Schouten J , Verschuur CVM , et al. Levodopa response in patients with early Parkinson disease: further observations of the LEAP study. Neurology 2023;100(4):e367–e376. 10.1212/WNL.0000000000201448. 36253105

[3] Connolly BS , Lang AE . Pharmacological treatment of Parkinson disease: a review. JAMA 2014;311(16):1670–1683. 10.1001/jama.2014.3654. 24756517

[4] Bryant MS , Rintala DH , Hou JG , et al. Gait variability in Parkinson's disease: influence of walking speed and dopaminergic treatment. Neurol Res 2011;33(9):959–964. 10.1179/1743132811Y.0000000044. 22080998 PMC5361771

[5] Bryant MS , Rintala DH , Hou JG , Lai EC , Protas EJ . Effects of levodopa on forward and backward gait patterns in persons with Parkinson's disease. NeuroRehabilitation 2011;29(3):247–252. 10.3233/NRE-2011-0700. 22142758 PMC3391536

[6] Curtze C , Nutt JG , Carlson‐Kuhta P , Mancini M , Horak FB . Levodopa is a double‐edged sword for balance and gait in people with Parkinson's disease. Mov Disord 2015;30(10):1361–1370. 10.1002/mds.26269. 26095928 PMC4755510

[7] Rochester L , Baker K , Nieuwboer A , Burn D . Targeting dopa‐sensitive and dopa‐resistant gait dysfunction in Parkinson's disease: selective responses to internal and external cues. Mov Disord 2011;26(3):430–435. 10.1002/mds.23450. 21462258

[8] Blin O , Ferrandez AM , Pailhous J , Serratrice G . Dopa‐sensitive and dopa‐resistant gait parameters in Parkinson's disease. J Neurol Sci 1991;103(1):51–54. 10.1016/0022-510x(91)90283-d. 1865232

[9] Fabbri M , Coelho M , Abreu D , et al. Do patients with late‐stage Parkinson's disease still respond to levodopa? Parkinsonism Relat Disord 2016;26:10–16. 10.1016/j.parkreldis.2016.02.021. 26972527

[10] Koller WC . Pharmacologic treatment of parkinsonian tremor. Arch Neurol 1986;43(2):126–127. 10.1001/archneur.1986.00520020020009. 3947248

[11] Fahn S , Oakes D , Shoulson I , et al. Levodopa and the progression of Parkinson's disease. N Engl J Med 2004;351(24):2498–2508. 10.1056/NEJMoa033447. 15590952

[12] Alberts JL , Rosenfeldt AB , Lopez‐Lennon C , Suttman E , Jansen AE , Imrey PB , Dibble LE . Effectiveness of a long‐term, home‐based aerobic exercise intervention on slowing the progression of Parkinson disease: Design of the cyclical lower extremity exercise for Parkinson disease II (CYCLE‐II) study. Phys Ther 2021;101(11):1‐10. 10.1093/ptj/pzab191. PMC8632855 34363478

[13] Eng JJ , Dawson AS , Chu KS . Submaximal exercise in persons with stroke: test‐retest reliability and concurrent validity with maximal oxygen consumption. Arch Phys Med Rehabil 2004;85(1):113–118. 10.1016/s0003-9993(03)00436-2. 14970978 PMC3167868

[14] Steffen T , Seney M . Test‐retest reliability and minimal detectable change on balance and ambulation tests, the 36‐item short‐form health survey, and the unified Parkinson disease rating scale in people with parkinsonism. Phys Ther 2008;88(6):733–746. 10.2522/ptj.20070214. 18356292

[15] Committee on Proficiency Standards for Clinical Pulmonary Function Laboratories . ATS statement: guidelines for the six‐minute walk test. Am J Respir Crit Care Med 2002;166(1):111–117. 10.1164/ajrccm.166.1.at1102. 12091180

[16] Hass CJ , Bishop M , Moscovich M , et al. Defining the clinically meaningful difference in gait speed in persons with Parkinson disease. J Neurol Phys Ther 2014;38(4):233–238. 10.1097/NPT.0000000000000055. 25198866

[17] Goetz CG , Tilley BC , Shaftman SR , et al. Movement Disorder Society‐sponsored revision of the Unified Parkinson's Disease Rating Scale (MDS‐UPDRS): scale presentation and clinimetric testing results. Mov Disord 2008;23(15):2129–2170. 10.1002/mds.22340. 19025984

[18] Mathias S , Nayak US , Isaacs B . Balance in elderly patients: the “get‐up and go” test. Arch Phys Med Rehabil 1986;67(6):387–389. 3487300

[19] Nyholm D , Jost WH . An updated calculator for determining levodopa‐equivalent dose. Neurol Res Pract 2021;3(1):58. 10.1186/s42466-021-00157-6. 34689840 PMC8543803

[20] Schade S , Mollenhauer B , Trenkwalder C . Levodopa equivalent dose conversion factors: an updated proposal including opicapone and safinamide. Mov Disord Clin Pract 2020;7(3):343–345. 10.1002/mdc3.12921. 32258239 PMC7111582

[21] Tomlinson CL , Stowe R , Patel S , Rick C , Gray R , Clarke CE . Systematic review of levodopa dose equivalency reporting in Parkinson's disease. Mov Disord 2010;25(15):2649–2653. 10.1002/mds.23429. 21069833

[22] Smulders K , Dale ML , Carlson‐Kuhta P , Nutt JG , Horak FB . Pharmacological treatment in Parkinson's disease: effects on gait. Parkinsonism Relat Disord 2016;31:3–13. 10.1016/j.parkreldis.2016.07.006. 27461783 PMC5048566

[23] Watts RL , Jankovic J , Waters C , Rajput A , Boroojerdi B , Rao J . Randomized, blind, controlled trial of transdermal rotigotine in early Parkinson disease. Neurology 2007;68(4):272–276. 10.1212/01.wnl.0000252355.79284.22. 17202432

[24] Postuma RB , Berg D , Stern M , et al. MDS clinical diagnostic criteria for Parkinson's disease. Mov Disord 2015;30(12):1591–1601. 10.1002/mds.26424. 26474316

[25] Pirker W , Katzenschlager R , Hallett M , Poewe W . Pharmacological treatment of tremor in Parkinson's disease revisited. J Parkinsons Dis 2023;13(2):127–144. 10.3233/JPD-225060. 36847017 PMC10041452

[26] Steffen TM , Hacker TA , Mollinger L . Age‐ and gender‐related test performance in community‐dwelling elderly people: Six‐Minute Walk Test, Berg Balance Scale, Timed Up & Go Test, and gait speeds. Phys Ther 2002;82(2):128–137. 10.1093/ptj/82.2.128. 11856064

[27] van der Zee S , Kanel P , Gerritsen MJJ , et al. Altered cholinergic innervation in de novo Parkinson's disease with and without cognitive impairment. Mov Disord 2022;37(4):713–723. 10.1002/mds.28913. 35037719 PMC9306739

[28] Karachi C , Grabli D , Bernard FA , et al. Cholinergic mesencephalic neurons are involved in gait and postural disorders in Parkinson disease. J Clin Invest 2010;120(8):2745–2754. 10.1172/JCI42642. 20628197 PMC2912198

[29] Paredes‐Rodriguez E , Vegas‐Suarez S , Morera‐Herreras T , De Deurwaerdere P , Miguelez C . The noradrenergic system in Parkinson's disease. Front Pharmacol 2020;11:435. 10.3389/fphar.2020.00435. 32322208 PMC7157437

[30] Devos D , Defebvre L , Bordet R . Dopaminergic and non‐dopaminergic pharmacological hypotheses for gait disorders in Parkinson's disease. Fundam Clin Pharmacol 2010;24(4):407–421. 10.1111/j.1472-8206.2009.00798.x. 20163480

[31] Fling BW , Cohen RG , Mancini M , Nutt JG , Fair DA , Horak FB . Asymmetric pedunculopontine network connectivity in Parkinsonian patients with freezing of gait. Brain 2013;136(Pt 8):2405–2418. 10.1093/brain/awt172. 23824487 PMC3722352

[32] Youn J , Lee JM , Kwon H , Kim JS , Son TO , Cho JW . Alterations of mean diffusivity of pedunculopontine nucleus pathway in Parkinson's disease patients with freezing of gait. Parkinsonism Relat Disord 2015;21(1):12–17. 10.1016/j.parkreldis.2014.10.003. 25455691

[33] Su ZH , Patel S , Gavine B , et al. Deep brain stimulation and levodopa affect gait variability in Parkinson disease differently. Neuromodulation 2023;26(2):382–393. 10.1016/j.neurom.2022.04.035. 35562261

[34] Fasano A , Aquino CC , Krauss JK , Honey CR , Bloem BR . Axial disability and deep brain stimulation in patients with Parkinson disease. Nat Rev Neurol 2015;11(2):98–110. 10.1038/nrneurol.2014.252. 25582445

[35] Duncan RP , Combs‐Miller SA , McNeely ME , et al. Are the average gait speeds during the 10 meter and 6 minute walk tests redundant in Parkinson disease? Gait Posture 2017;52:178–182. 10.1016/j.gaitpost.2016.11.033. 27915221 PMC5337136

